# Chimpanzee lip-smacking facilitates cooperative behaviour

**DOI:** 10.1038/srep13460

**Published:** 2015-08-21

**Authors:** Pawel Fedurek, Katie E. Slocombe, Jessica A. Hartel, Klaus Zuberbühler

**Affiliations:** 1 Institute of Biology, University of Neuchâtel, Switzerland; 2 Department of Psychology, University of York, Heslington, York, YO10 5DD, UK, UK; 3 Centre for Biocultural History, Aarhus University, Denmark; 4 Budongo Conservation Field Station, Masindi, Uganda; 5 School of Psychology and Neuroscience, University of St Andrews, Scotland (UK)

## Abstract

Signalling plays an important role in facilitating and maintaining affiliative or cooperative interactions in social animals. Social grooming in primates is an example of an interaction that requires coordination between partners but little is known about communicative behaviours facilitating this activity. In this study, we analysed the communication of wild chimpanzees of Budongo Forest, Uganda, as they entered and maintained a naturally occurring cooperative interaction: social grooming. We found that lip-smacking, a distinct multimodal oral gesture produced during grooming, coordinated this activity. Lip-smacking at the beginning of grooming bouts was significantly more often followed by longer and reciprocated bouts than silent grooming initiations. Lip-smacks were more likely to be produced when the risk of termination of the interaction by the recipient was high, for instance when grooming vulnerable body parts. Groomers were also more likely to produce lip-smacks during face-to-face grooming where the visual aspect of the signal could be perceived. Data are consistent with the hypothesis that chimpanzee lip-smacks function to coordinate and prolong social grooming, suggesting that this oral signal is an example of a communicative behaviour facilitating cooperative behaviour in chimpanzees.

Advertising behavioural intentions or subsequent behaviour is essential in initiating and maintaining friendly or cooperative interactions in animals^1^. This especially applies to interactions involving close physical proximity, which brings about vulnerability to potential aggression. As a consequence, many animals exhibit behaviours that have evolved specifically to signal a non-aggressive or benign attitude prior to or during affiliative or cooperative interactions. For example, play-bows in canids and play-faces in primates are produced to facilitate and maintain playful interactions with conspecifics^2^,^3^. Other examples are bowing or fluffing the head and neck to facilitate allo-preening in birds^4^ or tactile dance in cleaner fish and rocking dance in cleaner shrimps to advertise cleaning services to clients^5^,^6^.

In many primate species, lip-smacking, defined as the rapid closing and opening of the mouth and lips^7^, facilitates tolerance and affiliative interactions^8^. Baboons and macaques, for example, often produce lip-smacks during friendly approaches or face-to-face greetings^8^,^9^, as well as during mother-infant interactions^10^,^11^. Lip-smacking is a multimodal signal with a clear audible and visual element and the visual component has been shown to be sufficient in rhesus macaques to elicit reciprocation of this signal^12^. Lip-smacking in primates is also often associated with social grooming^9^,^13^. Grooming is an important behaviour in primates fulfilling several functions. For example, grooming plays an essential hygienic role in removing parasites and dirt from the hair and skin^14^,^15^. Grooming is also socially vital in reducing tension, promoting tolerance, and restoring relationships after aggression^16^,^17^,^18^. Finally, grooming facilitates coalitions and is crucial in establishing and maintaining social bonds^19^,^20^,^21^. At the same time, grooming is costly since it requires risky close physical contact between the two partners^22^, impairs vigilance^23^,^24^,^25^, prevents both partners from engaging in other activities^26^,^27^, and increases the risk of transmission of internal parasites^28^. Nevertheless, primates spend much of their social time grooming^19^, which, especially in chimpanzees, is often reciprocated by taking turns in coordinated ways^29^. Grooming therefore meets the criteria of a cooperative act^30^ defined as a social interaction that, regardless of short-term costs, increases fitness^31^.

To maximise the benefits of grooming, signals advertising grooming commitment and encouraging partners to engage in this interaction or preventing them from terminating it, would be beneficial. To date, however, little is known about the role of communication in instigating and maintaining grooming. Lip-smacking is a good candidate to function in this manner, since it is often associated with grooming. This especially applies to chimpanzees, which, in contrast to monkey species, produce lip-smacks almost exclusively in grooming contexts. In chimpanzees lip-smacks are produced by the groomer^32^ when initiating or during grooming bouts^33^,^34^, suggesting it plays a role in coordinating this social interaction. However, virtually no studies have systematically explored the role of lip-smacking in grooming, especially whether and how it facilitates the occurrence and maintenance of grooming interactions.

The aim of our study was to investigate the role of lip-smacking in coordinating dyadic grooming bouts in chimpanzees. We hypothesised that lip-smacks produced by the groomer function to initiate and prolong grooming bouts and to facilitate grooming reciprocation within the bout. As such, we predicted that grooming bouts would be longer if lip-smacks were produced at the start of a grooming interaction compared to bouts where these signals were not used. We also predicted that the groomee would be more likely to reciprocate grooming if lip-smacks were given by the groomer, and that groomers would be more likely to produce lip-smacks when engaging in higher-risk grooming of vulnerable body parts, to signal benign intent and to prevent early termination of the grooming bout by the recipient (e.g.,^22^,^35^). Since grooming is a valuable social commodity in chimpanzee society and vital for building alliances, we predicted that groomers would be more likely to use this signal when involved in particularly valuable interactions, such as when grooming preferred social partners (PSPs) or higher ranking individuals. Finally, since lip-smacking is a multimodal signal, we predicted that lip-smacking would be more frequent when partners were facing each other and recipients could detect the visual component of the signal (e.g.,^36^).

## Results

### Lip-smacking rates

Lip-smacks occurred in 65% of grooming bouts. In 54% of grooming bouts lip-smacks were produced during the first 10 s of the bout. Lip-smacking occurred in 41% of all 10 s samples taken from all groomers (47% in reciprocated and 35% in unreciprocated grooming bouts). Although there was some variation among the focal males in terms of the rate with which they produced lip-smacks (Mean = 45% of samples, SD = 18%), all of them produced lip-smacks (Min = 17% of samples, Max = 80%).

### Lip-smacking is associated with longer grooming bouts

**Figure 1:**
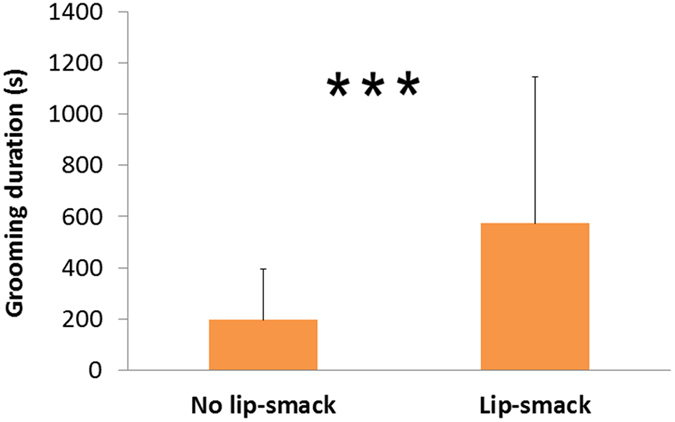
The relationship between lip-smacking and grooming bout duration (LMM, ***P < 0.001; Random effects: Groomer ID and Groomee ID; Error bars represent 1 SD).

Grooming bouts with lip-smacks given by the groomer in the first 10 s of the bout were significantly longer than those in which no lip-smacks occurred during the same period (β ± SE = 235.81 ± 71.79, z = 3.28, P = 0.001, Fig. 1). Since reciprocated grooming bouts were longer than unreciprocated ones (β ± SE = 515.30 ± 80.66, z = 6.39, P < 0.001), we tested whether lip-smacking predicted grooming duration in both reciprocated and unreciprocated grooming bouts. Both unreciprocated (β ± SE = 194.47 ± 55.42, z = 3.51, P < 0.001) and, although not significantly (β ± SE = 441.65 ± 240.44, z = 1.84, P = 0.066), reciprocated grooming bouts were longer if there was a lip-smack during the first 10 s of the bout.

### Lip-smacking is associated with reciprocated grooming bouts

**Figure 2:**
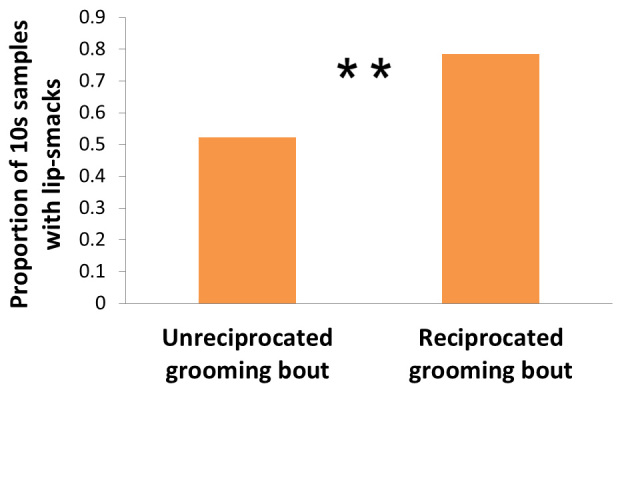
The relationship between lip-smacking within the first 10 s of a grooming bout and whether or not the bout was reciprocated (GLMM, **P < 0.01; Random effects: Groomer ID and Groomee ID).

A grooming bout was more likely to be reciprocated than unreciprocated if lip-smacks were given during the first 10 s of a bout (β ± SE = 1.44 ± 0.54, z = 2.70, P = 0.007; Fig. 2). Lip-smacking was a better predictor of reciprocity than whether or not partners were PSPs (β ± SE = −0.78 ± 0.64, z = −1.21, P = 0.225) or rank distance between them (β ± SE = −0.00 ± 0.00, z = −1.34, P = 0.180).

### Lip-smacks are more likely to be given when grooming vulnerable body parts and when the recipient can see the groomer

**Figure 3:**
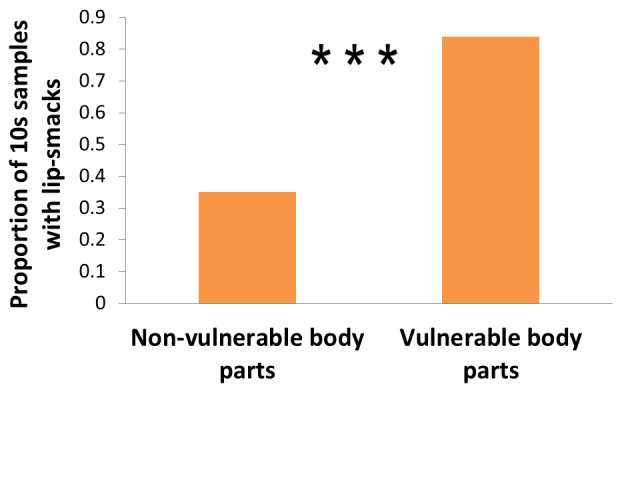
The relationship between lip-smacking and whether or not the groomed part was a vulnerable body part (GLMM, ***P < 0.001; Random effects: Groomer ID, Groomee ID and Grooming bout ID).

Lip-smacks were more likely to be produced if grooming vulnerable body parts, such as the head and ano-genital areas, than non-vulnerable body parts (Table 1, Fig. 3). Lip-smacks were also more likely to be given if groomers were in front of the groomees (54% of samples taken throughout grooming bouts contained lip-smacks) than when they were oriented in other ways (34% of samples contained lip-smacks; Table 1). Contrary to our predictions, however, males were not more likely to give lip-smacks when grooming PSPs or higher ranking individuals (Table 1).

## Discussion

A lot of research has highlighted the social importance of reciprocal grooming in chimpanzees and other primates^26^,^37^,^38^ but little is known about how primates communicate to facilitate this cooperative behaviour. In monkey species, employing specific body postures or presenting body parts seem to solicit or demand more grooming from the partner^35^,^39^. Similarly, it has been suggested that in chimpanzees self-scratching is used to solicit grooming or to request grooming of specific body parts^40^,^41^,^42^. However, to our knowledge, this is the first study to identify a signal produced by the groomer that seems to prolong grooming bouts and promote within-bout reciprocity, which both apparently increase the social value of the interaction^26^,^37^,^43^. Chimpanzee grooming bouts can last for considerable amounts of time, suggesting that they require coordination between partners’ activities. Our data suggest that lip-smacks function in this way by reducing the probability of early termination by the recipient and by increasing the likelihood of reciprocity.

Individuals reliably produced lip-smacks when grooming vulnerable body parts, such as the partner’s head and ano-genital area. Most likely, groomers produced the signal to communicate benign intent in such socially ‘risky’ situations when the probability of premature termination was highest. This finding is in line with a study on vervet monkeys showing that lip-smacking is more likely to occur in potentially stressful grooming situations, such as shortly before using the mouth to groom or if grooming mothers with infants, probably to avoid grooming termination^35^. Such ‘reassuring’ signals during friendly or cooperative interactions also occur in other social animals. For example, bowing during social playing in canids are often produced before or after play biting probably because biting is normally associated with aggression and therefore could be misinterpreted by the partner^3^. Lip-smacking in grooming contexts seems to be an example of such a signal maintaining cooperative interaction in socially risky situations in primates.

Lip-smacking was more likely to occur when the groomer was positioned in sight rather than out of sight of the groomee. Lip-smacking is a multimodal signal with clear audible output combined with salient facial movements and signallers seemed more likely to use the signal when the recipient could not only hear but also see the signal. This is consistent with the fact that in monkey species lip-smacks are often produced in face-to-face interactions^9^. Sensitivity to the visual attention of the recipient when producing signals with a visual component has commonly been argued to be characteristic of ape gesture production, and in this context such flexible use of signals has been widely used as a marker of intentional signal production^44^,^45^.

Finally, our data do not support the hypothesis that lip-smacking is more frequently produced when grooming well-affiliated or higher-ranking individuals. One of the reasons for this may be that the social relationships, including the dominance position and affiliative relationships, between the Sonso males were unstable at the time of this study (P. Fedurek, unpublished data). Therefore, the lack of stable friendship patterns may have reduced the chances of seeing a clear pattern between the occurrence of lip-smack signals and the level of affiliation between the partners. It is also possible that, during times of social instability, it pays individuals to use affiliative signals to neutral social partners or lower ranking males to cultivate potential future allies. Alternatively, lip-smacking may be a flexible short-term affiliative signal employed between both preferred and neutral social partners in a similar way as pant hoot chorusing^46^.

Overall, our data suggest that chimpanzees flexibly modulate the production of lip-smacks, which in turn seems to influence the social nature of grooming bouts by making them longer and reciprocated. In this respect, lip-smacking might be interpreted as a signal that facilitates cooperative acts in chimpanzees, with similarities to how language facilitates and coordinates joint activities in humans. Indeed, on a proximate level, lip-smacking, although unvoiced, requires some control over the supra-laryngeal parts of the vocal tract and rhythmic facial expressions^7^,^47^ that are similar to human speech production. This has led some scientists to interpret lip-smacking as a candidate precursor to speech signals^7^,^48^.

In conclusion, our study suggests that chimpanzee lip-smacking functions to maintain and prolong grooming bouts, as well as to facilitate within-bout reciprocity. Lip-smacks seem to encourage the recipient to engage in this activity and this coordination of the activities of the interactants is critical to the occurrence of long, reciprocal and thus socially valuable grooming bouts. Lip-smacking might therefore be an example of an oral signal that facilities cooperative behaviour in chimpanzees.

## Methods

### Study site and study subject

We studied the Sonso community of the Budongo Forest Reserve, Uganda. At the time of the study, the community contained 75 individuals with a core range of around 15 km^2^. Study subjects were adults (N = 11 males: ≥ 16 years; N = 24 females: ≥ 15 years^49^) and adolescents (N = 3 early males: 8–12 years; N = 3 late males: 13–15 years; N = 9 early females: 8–10 years old; N = 4 late females: 11–14 years).

### Data collection and definitions

This study was approved by, and carried out in accordance with, the Department of Psychology Ethics Committee at the University of York. The study was approved by the Uganda Wildlife Authority and the Uganda National Council for Science and Technology.

#### Lip-smacking and grooming

The study was conducted between May and October 2013 and between January and September 2014. Focal animal sampling^50^ was the method of data collection and a randomly chosen adult or late adolescent male was followed between 7:00 and 16:30. To ensure accuracy of data collection on this subtle signal, data were only collected on grooming bouts where the focal animal was between 5 and 7 m from the observer, the grooming was with only one partner, and no other grooming bouts were taking place within 5 m of the focal animal.

Grooming was defined as manually picking through the hair of a partner to remove items, such as parasites or clean small injures^30^. A grooming bout was defined as a period of grooming that was separated by at least 1 min of other activities (including resting). Reciprocated grooming was defined as a grooming bout where the two partners switched roles, while unreciprocated grooming was when only one partner engaged in grooming.

**Figure 4:**
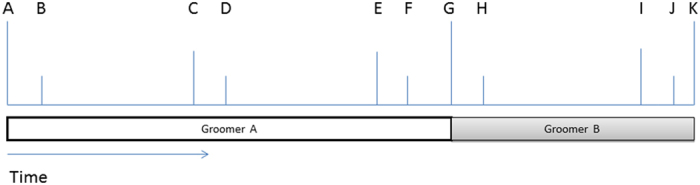
The outline of the elements within a grooming bout used during data collection and analysis. A-K: grooming bout; A: start of a grooming bout; K: end of a grooming bout; G: role reversal; A-C, C-E: complete one-minute periods of individual A grooming individual B; G-I complete one-minute period of individual B grooming individual A; A-B, C-D, E-F: 10 s periods sampled of individual A’s behaviour as groomer for the analyses; G-H, I-J: 10 s periods sampled of individual B’s behaviour as groomer for the analyses.

When the focal animal was involved in grooming with another individual we recorded the time (in seconds) of the start and end of a grooming bout, the identity of the individual initiating a grooming bout and the identity of the partner. To minimise the problem of temporal non-independence of the data-points, we recorded details on grooming and lip-smacking from the first 10 s of every minute during the bout (Fig. 4). During these 10 s periods of a grooming bout, we recorded information including: (i) the identity of the individuals providing and receiving grooming, (ii) whether or not (0/1) the provider of grooming lip-smacked, (iii) whether or not the groomer was in front of the groomee (0/1) and (iv) the body part (i.e., the head, ano-genital areas, the chest/front, the back and the limbs) that was being groomed. Since only providers but not receivers of grooming lip-smacked (and there was no reciprocation of groomers’ lip-smacks by groomees), all data on lip-smacking were collected from providers of grooming. We classified the head and ano-genital areas (i.e., sensitive body areas containing vital organs, where receiving aggression could result in life threatening or debilitating injuries) as vulnerable body parts, whereas the chest, the back and the limbs as non-vulnerable body parts. If during the sampled 10 s period the groomer switched from grooming one body part to another, we recorded the body part that was being groomed longer. After a role reversal, we collected data during the first 10 s following the role reversal, followed by further 10 s samples throughout the following 1 min blocks (Fig. 4).

#### Dataset

Our data set comprised of a total of 1385 10 s samples taken from 192 grooming bouts (of which 56 were reciprocated and 136 unreciprocated). We omitted in the analyses the 10 s samples (N = 347 of 1385) where two individuals were grooming each other simultaneously since it was difficult to reliably establish whether both or only one grooming partner was lip-smacking. Of the remaining 1038 10 s samples (501 from reciprocated and 537 from unreciprocated grooming bouts) that were entered into analyses, 748 of these samples from 129 bouts were provided by focal males when grooming other adult or late adolescent males, 199 samples from 35 bouts by focal males when grooming females or early adolescent males, and 91 samples from 28 bouts by non-focal individuals (females and early adolescent males).

#### Preferred social partners

PSPs were identified only for adult and late adolescent males. PSPs were established on the basis of three different dyadic association measures^51^ (see Supplementary Information).

#### Dominance status

Dominance status was established only for adult and late adolescent males, using the Elo-rating procedure^52^ (see Supplementary Information). Dominance distance between two grooming partners was established by deducting the Elo-rating score of the groomee from the Elo-rating score of the groomer.

### Statistical analysis

Linear Mixed-Effect Models (LMM) and Generalised Linear Mixed-Effect Models (GLMM) were used in statistical analyses. In the majority of the analyses the first 10 s within every minute of a grooming bout was set as a single data-point. In our models the identities of both the provider and receiver of grooming for every data-point were put as random effects. Since grooming bouts often generated more than one data-point, we put the identity of a grooming bout as an additional random effect. All statistical analyses were conducted using STATA 12.0 software (StataCorp LP, College Station, TX, USA).

#### Models created

To investigate whether grooming bouts in which there was a lip-smack at their onset were longer than those in which there was not, we created a LMM in which we put as the dependent variable grooming bout duration (in seconds) and as the independent variables whether or not (0/1) lip-smacking occurred during the first 10 s of the grooming bout and whether or not (0/1) the grooming bout was reciprocated. In this analysis a grooming bout was a single data-point and data from all grooming bouts were included (N = 192). Groomer ID and groomee ID were put as random effects.

To investigate whether lip-smacking at the onset of a grooming bout predicted whether or not a grooming bout was reciprocated, we created another model in which as the dependent variable we used the type of a grooming bout (reciprocated (1) and unreciprocated (0)) whereas as the independent variable we put whether or not (0/1) there was a lip-smack during the first 10 s of that bout. We also inserted into the model as the potential confounding independent variables, whether or not the groomee was a PSP to the groomer (0/1) and the dominance distance between the two grooming partners (in Elo-rating scores). Since we had data on affiliative and dominance relationships only for adult and late adolescent males, we conducted this analysis only for this age/sex category. In this analysis, a grooming bout was a single data-point (N = 129 grooming bouts). Groomer ID and Groomee ID were put as random effects.

To test which factors affect the probability of the groomer producing lip-smacks, we created a GLMM in which we put as the dependent variable whether or not (0/1) the groomer lip-smacked in the first 10 s of every minute, and as the independent variables the dominance status distance between the grooming partners (in Elo-rating scores), whether or not (0/1) the groomee was a PSP to the groomer, whether or not (0/1) the groomer was in a direct visual contact with the groomee, as well as whether or not (0/1) vulnerable body parts were being groomed. Again, since we had data on PSPs and dominance statuses only for adult and late adolescent males, only samples from grooming bouts between these males were analysed in this model (N = 748 10 s samples from 129 grooming bouts). The ID of the groomer, the ID of the groomee and the ID of the grooming bout were put as random effects.

## Additional Information

**How to cite this article**: Fedurek, P. *et al.* Chimpanzee lip-smacking facilitates cooperative behaviour. *Sci. Rep.* **5**, 13460; doi: 10.1038/srep13460 (2015).

## Supplementary Material

Supplementary Information

## Figures and Tables

**Table 1:** The relationship between lip-smacking and various independent variables.

**Independent variables**	**Coefficient**	**SE**	**z value**	**P value**	**95% confidence interval**	
Dominance distance	0.00	0.00	0.44	0.659	−0.00	0.00
Affiliation level (PSP or Non-PSP)	0.03	0.31	0.11	0.914	−0.57	0.64
Groomer in front/behind groomee	0.50	0.23	2.16	0.031	0.05	0.96
Vulnerable/non-vulnerable body part	2.12	0.39	5.48	<0.001	1.36	2.88

GLMM; Dependent variable: lip-smacking (0/1); Random effects: Groomer ID, Groomee ID and Grooming bout ID. Grooming bouts between adult and late adolescent males only (N = 748 data-points from 129 grooming bouts).

## References

[b1] Smith W. J. The behavior of communicating: an ethological approach. (Harvard University Press, 1977).

[b2] Flack J. C., Jeannotte L. A. & de Waal F. B. M. Play signaling and the perception of social rules by juvenile chimpanzees (Pan troglodytes). Journal of Comparative Psychology 118, 149–159, 10.1037/0735-7036.118.2.149 (2004). 15250802

[b3] Bekoff M. Play signals as punctuation: the structure of social play in canids. Behaviour 132, 419–429, 10.1163/156853995×00649 (1995).

[b4] Katzir G. Bowing and allopreening of captive Jackdaws Corvus monedula. Ibis 125, 516–523, 10.1111/j.1474-919X.1983.tb03145.x (1983).

[b5] Becker J. H . A., Curtis L. M. & Grutter A. S. Cleaner Shrimp Use a Rocking Dance to Advertise Cleaning Service to Clients. Current Biology 15, 760–764, doi: (http://dx.doi.org/10.1016/j.cub.2005.02.067 2005). 15854910 10.1016/j.cub.2005.02.067

[b6] Grutter A. S. Cleaner fish use tactile dancing behavior as a preconflict management strategy. Current Biology 14, 1080–1083, http://dx.doi.org/10.1016/j.cub.2004.05.048 (2004). 15203000 10.1016/j.cub.2004.05.048

[b7] Ghazanfar A. A., Takahashi D. Y., Mathur N. & Fitch W. T. Cineradiography of monkey lip-smacking reveals putative precursors of speech dynamics. Current Biology 22, 1176–1182 (2012). 22658603 10.1016/j.cub.2012.04.055 PMC3569518

[b8] van Hooff J. A. R. A. M. in Primate ethology (ed Morris D. ) 7–68 (Aldine, 1967).

[b9] van Hooff J. A. R. A. M. Facial expressions of higher primates. Symp. Zool. Soc. Lond. 8, 97–125 (1962).

[b10] Mowbray J. B. & Cadell T. E. Early behavior patterns in rhesus monkeys. Journal of Comparative and Physiological Psychology 55, 350–357, 10.1037/h0041420 (1962). 14476741

[b11] Ferrari P. F., Paukner A., Ionica C. & Suomi S. J. Reciprocal face-to-face communication between rhesus macaque mothers and their newborn infants. Current Biology 19, 1768–1772, http://dx.doi.org/10.1016/j.cub.2009.08.055 (2009). 19818617 10.1016/j.cub.2009.08.055 PMC2784245

[b12] Ghazanfar A. A., Morrill R. J. & Kayser C. Monkeys are perceptually tuned to facial expressions that exhibit a theta-like speech rhythm. Proceedings of the National Academy of Sciences 110, 1959–1963, 10.1073/pnas.1214956110 (2013). PMC3562783 23319616

[b13] Maestripieri D. Gestural Communication in Macaques: Usage and Meaning of Nonvocal Signals. Evolution of Communication 1, 193–222, 10.1075/eoc.1.2.03mae (1997).

[b14] Akinyi M. Y. *et al.* Role of grooming in reducing tick load in wild baboons (Papio cynocephalus). Animal Behaviour 85, 559–568, http://dx.doi.org/10.1016/j.anbehav.2012.12.012 (2013). 24659824 10.1016/j.anbehav.2012.12.012 PMC3961061

[b15] Hutchins M. & Barash D. P. Grooming in primates: Implications for its utilitarian function. Primates 17, 145–150, 10.1007/bf02382848 (1976).

[b16] Terry R. L. Primate grooming as a tension reduction mechanism. The Journal of Psychology 76, 129–136, 10.1080/00223980.1970.9916830 (1970). 4989995

[b17] Aureli F., Cords M. & van Schaik C. P. Conflict resolution following aggression in gregarious animals: a predictive framework. Animal Behaviour 64, 325–343, http://dx.doi.org/10.1006/anbe.2002.3071 (2002).

[b18] Barrett L., Gaynor D. & Henzi S. P. A dynamic interaction between aggression and grooming reciprocity among female chacma baboons. Animal Behaviour 63, 1047–1053, http://dx.doi.org/10.1006/anbe.2002.3008 (2002).

[b19] Dunbar R. I. M. Functional significance of social grooming in primates. Folia Primatologica 57, 121–131 (1991).

[b20] Seyfarth R. M. & Cheney D. L. Grooming, alliances and reciprocal altruism in vervet monkeys. Nature 308, 541–543, 10.1038/308541a0 (1984). 6709060

[b21] Dunbar R. I. M. The social role of touch in humans and primates: Behavioural function and neurobiological mechanisms. Neuroscience and Biobehavioral Reviews 34, 260–268, 10.1016/j.neubiorev.2008.07.001 (2010). 18662717

[b22] Moser R., Cords M. & Kummer H. Social influences on grooming site preferences among captive long-tailed macaques. International Journal of Primatology 12, 217–230, 10.1007/BF02547585 (1991).

[b23] Maestripieri D. Vigilance costs of allogrooming in macaque mothers. Am. Nat. 141, 744–753, 10.1086/285503 (1993). 19426008

[b24] Cords M. Predator vigilance costs of allogrooming in wild blue monkeys. Behaviour 132, 559–569 (1995).

[b25] Mooring M. S. & Hart B. L. Costs of allogrooming in impala: distraction from vigilance. Animal Behaviour 49, 1414–1416, http://dx.doi.org/10.1006/anbe.1995.0175 (1995).

[b26] Fedurek P. & Dunbar R. I. M. What does mutual grooming tell us about why chimpanzees groom? Ethology 115, 566–575, 10.1111/j.1439-0310.2009.01637.x (2009).

[b27] Dunbar R. I. M. & Sharman M. Is social grooming altruistic? Zeitschrift Fur Tierpsychologie 64, 163–173 (1984).

[b28] Rimbach R. *et al.* Brown spider monkeys (Ateles hybridus): a model for differentiating the role of social networks and physical contact on parasite transmission dynamics. Philos. Trans. R. Soc. B-Biol. Sci. 370, 10.1098/rstb.2014.0110 (2015). PMC4410376 25870396

[b29] Machanda Z. P., Gilby I. C. & Wrangham R. W. Mutual grooming among adult male chimpanzees: the immediate investment hypothesis. Animal Behaviour 87, 165–174, http://dx.doi.org/10.1016/j.anbehav.2013.10.028 (2014).

[b30] Hemelrijk C. & Steinhauser J. in Handbook of Paleoanthropology (eds Winfried Henke & Ian Tattersall ) Ch. 43, 1321–1346 (Springer Berlin Heidelberg, 2007).

[b31] Brown J. L. The evolution of behavior. (Norton, 1975).

[b32] Nishida T., Mitani J. C. & Watts D. P. Variable grooming behaviours in wild chimpanzees. Folia primatologica 75, 31–36, 10.1159/000073429 (2004). 14716152

[b33] de Waal F. in Chimpanzee cultures (eds Wrangham R., McGrew W. C., de Waal F., & Heltne P. G. ) 243–256 (Harvard University Press, 1996).

[b34] King B. J. The dynamic dance: nonvocal communication in African great apes. (Harvard University Press, 2004).

[b35] van de Waal E., Spinelli M., Bshary R., Ros A. & Noë R. Negotiations over grooming in wild vervet monkeys (Chlorocebus pygerythrus). International Journal of Primatology 34, 1153–1171, 10.1007/s10764-013-9729-1 (2013).

[b36] Yehia H. C., Kuratate T. & Vatikiotis-Bateson E. Linking facial animation, head motion and speech acoustics. Journal of Phonetics 30, 555–568, http://dx.doi.org/10.1006/jpho.2002.0165 (2002).

[b37] Mitani J. C. Male chimpanzees form enduring and equitable social bonds. Animal Behaviour 77, 633–640, 10.1016/j.anbehav.2008.11.021 (2009).

[b38] Silk J. *et al.* Female chacma baboons form strong, equitable, and enduring social bonds. Behav. Ecol. Sociobiol. 64, 1733–1747, 10.1007/s00265-010-0986-0 (2010). 20976293 PMC2952770

[b39] Tsukahara T. Initiation and solicitation in male-female grooming in a wild Japanese macaque troop on yakushima island. Primates 31, 147–156, 10.1007/BF02380937 (1990).

[b40] Pika S. & Mitani J. Referential gestural communication in wild chimpanzees (Pan troglodytes). Current Biology 16, R191–R192, 10.1016/j.cub.2006.02.037 (2006). 16546066

[b41] Nishida T. Chimpanzees of the Lakeshore. Natural history and culture at Mahale. (Cambridge University Press, 2012).

[b42] Hobaiter C. & Byrne R. W. The meanings of chimpanzee gestures. Current Biology 24, 1596–1600, http://dx.doi.org/10.1016/j.cub.2014.05.066 (2014). 24998524 10.1016/j.cub.2014.05.066

[b43] Silk J., Alberts S. & Altmann J. Social relationships among adult female baboons (Papio cynocephalus) II. Variation in the quality and stability of social bonds. Behav. Ecol. Sociobiol. 61, 197–204, 10.1007/s00265-006-0250-9 (2006).

[b44] Hostetter A. B., Cantero M. & Hopkins W. D. Differential use of vocal and gestural communication by chimpanzees (Pan troglodytes) in response to the attentional status of a human (Homo sapiens). Journal of Comparative Psychology 115, 337–343, 10.1037/0735-7036.115.4.337 (2001). 11824896 PMC2080764

[b45] Call J. & Tomasello M. in The gestural communication of apes and monkeys (eds Call J. & Tomasello M. ) 197–220 (Lawrence Erlbaum Associates, 2007).

[b46] Fedurek P., Machanda Z., Schel A. M. & Slocombe K. E. Pant hoot chorusing and social bonds in male chimpanzees. Animal Behaviour 86, 189–196 (2013).

[b47] Ghazanfar A. Multisensory vocal communication in primates and the evolution of rhythmic speech. Behav. Ecol. Sociobiol. 67, 1441–1448, 10.1007/s00265-013-1491-z (2013). PMC3821777 24222931

[b48] Lameira A. R., Maddieson I. & Zuberbühler K. Primate feedstock for the evolution of consonants. Trends in Cognitive Sciences 18, 60–62, http://dx.doi.org/10.1016/j.tics.2013.10.013 (2014). 24238780 10.1016/j.tics.2013.10.013

[b49] Goodall J. The chimpanzees of Gombe: patterns of behavior. (Harvard University Press, 1986).

[b50] Altmann J. Observational study of behaviour: sampling methods. Behaviour 49, 227–267, 10.1163/156853974×00534 (1974). 4597405

[b51] Gilby I. C. & Wrangham R. W. Association patterns among wild chimpanzees (Pan troglodytes schweinfurthii) reflect sex differences in cooperation. Behav. Ecol. Sociobiol. 62, 1831–1842, 10.1007/s00265-008-0612-6 (2008).

[b52] Neumann C. *et al.* Assessing dominance hierarchies: validation and advantages of progressive evaluation with Elo-rating. Animal Behaviour 82, 911–921 (2011).

