# Supplementary material for: Chimpanzee lip-smacking facilitates cooperative behaviour
Source: Sci Rep. 2015 Aug 21;5:13460. doi: 10.1038/srep13460 (PMC4544027; doi:10.1038/srep13460)
Supplement: Supplementary Information [file srep13460-s1.pdf]

Chimpanzee lip-smacking facilitates cooperative behaviour

Pawel Fedurek [a, d], Katie E. Slocombe [b], Jessica A. Hartel [c] & Klaus Zuberbühler [a,d,e]

[a] Institute of Biology, University of Neuchâtel, Switzerland; [b] Department of Psychology, University of York, UK; [c] Centre for Biocultural History, Aarhus University, Denmark; [d] Budongo Conservation Field Station, Masindi, Uganda; [e] School of Psychology and Neuroscience, University of St Andrews, Scotland (UK)

SUPPLEMENTARY INFORMATIONS

SUPPLEMENTARY METHODS

Male preferred social partners

PSPs were established on the basis of three different dyadic association measures. The first measure, Simple Ratio Index (SRI), reflects the total proportion of scans in which both individuals were together in the same party [1], or:

$$\mathrm{SRI}_{\mathrm{AB}} = \frac{P_{AB}}{P_A + P_B - P_{AB}}$$

Where P_{AB} = the number of parties containing both A and B, P_A = the number of parties containing A, P_B= the number of parties containing B.

The second dyadic association measure is the '5 metre association index' (5M) [2] which measures the frequency with which a dyad was observed within 5m of one anoth er, given that one of the individuals was present in the party and another one was a focal:

$$5\mathrm{M}_{\mathrm{AB}} = \frac{A_f(B_5) + B_f(A_5)}{A_f\left(B_p\right) + B_f(A_p)}$$

Where $A_f(B_5)$= the number of instances in which A was the focal animal and B was within 5m of A, $B_f(A_5)$= the number of instances in which B was the focal animal and A was within 5m, $A_f(B_p)$= the number of instances A was the focal and B was in the same party, $B_f(A_p)$= B was the focal and A was in the same party.

The third employed dyadic association measure is the ‘nearest neighbour association index’ (NN) [2], which reflects the frequency with which two individuals were observed as nearest neighbours, provided that one was the focal and the other was within 5m, or:

$$\mathrm{NN_{AB}} = \frac{A_f(B_{nn}) + B_f(A_{nn})}{A_f(B_5) + B_f(A_5)}$$

Where $A_f(B_{nn})$= the number of instances A was the focal and B was the nearest neighbour and $B_f(A_{nn})$= the number of instances B was the focal and A was the nearest neighbour.

To calculate PSPs for each focal chimpanzee, we first examined each index (SRI, 5N and NN) to identify any individuals for whom the index value was ½ standard deviation larger than the focal animal’s average (labelled as “associate”). In order to be classified as a PSP, individuals had to be categorised as an associate on at least two of the three indexes [2]. Since association dynamics in chimpanzees change on a temporal basis (e.g., [3]), we conducted association calculations for three separate periods with durations between four and five months: between June and October 2013, February and May 2014, and between June and September 2014. For the first, second and third study period we identified 28, 34 and 30 preferred social partners across the focal male chimpanzees, respectively. All remaining possible social partners for each focal were labelled as neutral social partners (non-PSPs). It is important to note that we calculated unidirectional rather than mutual PSPs, therefore, within a dyad A might be a preferred social partner for B, B however might not be a preferred social partner for A.

Dominance status

Dominance status was established only for adult and late adolescent males, using the Elo-rating procedure. This method is based on a sequence in which interactions between individuals occur rather than on an interaction matrix [4]. At the onset of the process each individual is given the same rating of a value 1000. After each agonistic or submissive interaction the score is updated with the winner of the interaction gaining whereas the looser losing points [4]. The number of points gained or lost by two interacting individuals is dependent on the expected outcome which in turn depends on previous interactions between these two individuals [5]. The scores were based on interactions such as pant grunts (i.e., vocalisations given by males to other males that outrank them) combined with the outcomes of dyadic win-lose agonistic interactions (i.e. physical attack, chase, charge, displacements, etc. [6-8]) recorded during the study period and, for the purpose of analyses, calculated for each day of data collection. The Elo-rating method has several advantages over more traditional methods such as sensitivity to short-term demography changes, effectiveness in tracking hierarchy dynamics on short-term scales, and more effective evaluation of relative hierarchy position between individuals with undecided interactions [4]. We believe that this method was especially effective in establishing dominance positions of the Sonso males, since the hierarchy was unstable throughout the study period with no clear alpha male after one of the males had lost his alpha status prior to the study period. Elo-rating scores were calculated using R v.3.1.1 (The R Foundation for Statistical Computing, Vienna, Austria, http://www.r-project.org).

SUPPLEMENTARY REFERENCES

1 Cairns, S. J. & Schwager, S. J. A comparison of association indexes. *Animal Behaviour* **35**, 1454-1469, doi:10.1016/s0003-3472(87)80018-0 (1987).

2 Gilby, I. C. & Wrangham, R. W. Association patterns among wild chimpanzees (Pan troglodytes schweinfurthii) reflect sex differences in cooperation. *Behav. Ecol. Sociobiol.* **62**, 1831-1842, doi:10.1007/s00265-008-0612-6 (2008).

3 Fedurek, P., Machanda, Z., Schel, A. M. & Slocombe, K. E. Pant hoot chorusing and social bonds in male chimpanzees. *Animal Behaviour* **86**, 189-196 (2013).

4 Neumann, C. *et al.* Assessing dominance hierarchies: validation and advantages of progressive evaluation with Elo-rating. *Animal Behaviour* **82**, 911-921, doi:http://dx.doi.org/10.1016/j.anbehav.2011.07.016 (2011).

5 Elo, A. E. *The Rating of Chess Players, Past and Present*. (Arco, 1978).

6 Goodall, J. *The chimpanzees of Gombe: patterns of Behavior*. (Harvard University Press, 1986).

7 Bygott, J. D. in *The great apes* (eds D.A. Hamburg & E.R. McCown) 405-428 (Benjamin/Cummings, 1989).

8 Muller, M. N. & Wrangham, R. W. Dominance, aggression and testosterone in wild chimpanzees: a test of the ' challenge hypothesis '. *Animal Behaviour* **67**, 113-123, doi:10.1016/j.anbehav.2003.03.013 (2004).
